# A Dilatometric Study of Tempering Complemented by Mössbauer Spectroscopy and other Characterization Techniques

**DOI:** 10.1038/s41598-017-17654-x

**Published:** 2017-12-11

**Authors:** I. Vieira, J. Klemm-Toole, E. Buchner, D. L. Williamson, K. O. Findley, E. De Moor

**Affiliations:** 1 Advanced Steel Processing and Products Research Center, Colorado School of Mines, 1500 Illinois St., Golden, CO 80401 USA; 2 Department of Physics, Colorado School of Mines, 1500 Illinois St., Golden, CO 80401 USA

## Abstract

A new approach for non-isothermal tempering analysis utilizing dilatometry is proposed and was carried out on a medium carbon steel with high silicon and additions of Mo and V for secondary hardening. The method includes a second non-isothermal step performed with the same heating rate (2 °C/min) used for the first step in order to create a baseline for analysis. The results were correlated with several other characterization techniques. Mössbauer spectroscopy confirmed the formation of transition carbides by auto-tempering as well as the presence of retained austenite decomposition (stage II) and cementite precipitation (stage III), which demonstrated significant overlap. Electrical resistivity measurements were correlated with dislocation densities obtained through X-ray diffraction analysis. Transmission electron microscopy dark field images confirmed the secondary hardening assessment from dilatometry.

## Introduction

The main mechanisms associated with martensite tempering are commonly described as a function of tempering temperature^1^. The various stages of tempering have been extensively investigated *e*.*g*.^1–4^. At temperatures lower than 100 °C, carbon segregation and clustering can be observed. The first stage is expected in the range of 100 to 250 °C with the formation of transition carbides. Between 200 and 300 °C, retained austenite transforms to ferrite and cementite, constituting the second stage. The third stage, associated with the precipitation of cementite, takes place between 250 and 350 °C. Around 400 °C recovery of the defect structure begins. During this process, dislocation density decreases, subgrain boundaries form and then begin to coarsen. Given enough time, the shape of the cementite becomes spheroidal. The recrystallization process happens at 600 to 700 °C and occurs more readily in steels with low carbon contents because the process is inhibited by the pinning action of carbides at the various martensite boundaries^4^. During recovery and recrystallization processes in a 500 to 700 °C temperature range, steels containing strong carbide forming elements, such as Cr, V, Mo and Nb, exhibit alloy carbide formation and as a consequence secondary hardening occurs, sometimes referred to as stage IV. Coarsening of the alloy carbides causes a decrease in hardness at high tempering temperatures.

Non-isothermal tempering analysis performed with dilatometry has been well established as a method to study stages I through III of tempering^5–9^. Stage IV has also been approached utilizing non-isothermal techniques, however less characterization was performed to correlate dilatometric analysis and the different tempering phenomena at high temperature (between 500 and 700 °C)^10–12^.

The current study proposes a different approach for non-isothermal tempering analysis and the technique is applied over the whole range of tempering temperatures. Vickers micro-hardness, Mössbauer spectroscopy, x-ray diffraction (XRD), electrical resistivity, and transmission electron microscopy (TEM) were utilized to help explain the response observed by dilatometry.

## Methods

The chemical composition of the medium carbon steel analyzed in the present study is listed in Table 1 in wt pct and at pct.

**Table 1:** Chemical Composition of the Investigated Steel.

	C	Mn	Si	Ni	Cr	Mo	V	Al	N	S	P	Fe
wt pct	0.40	1.42	1.49	1.20	0.01	0.25	0.25	0.035	0.018	0.005	0.010	94.9
at pct	1.81	1.40	2.88	1.11	0.01	0.14	0.27	0.070	0.070	0.008	0.018	92.2

**Figure 1:**
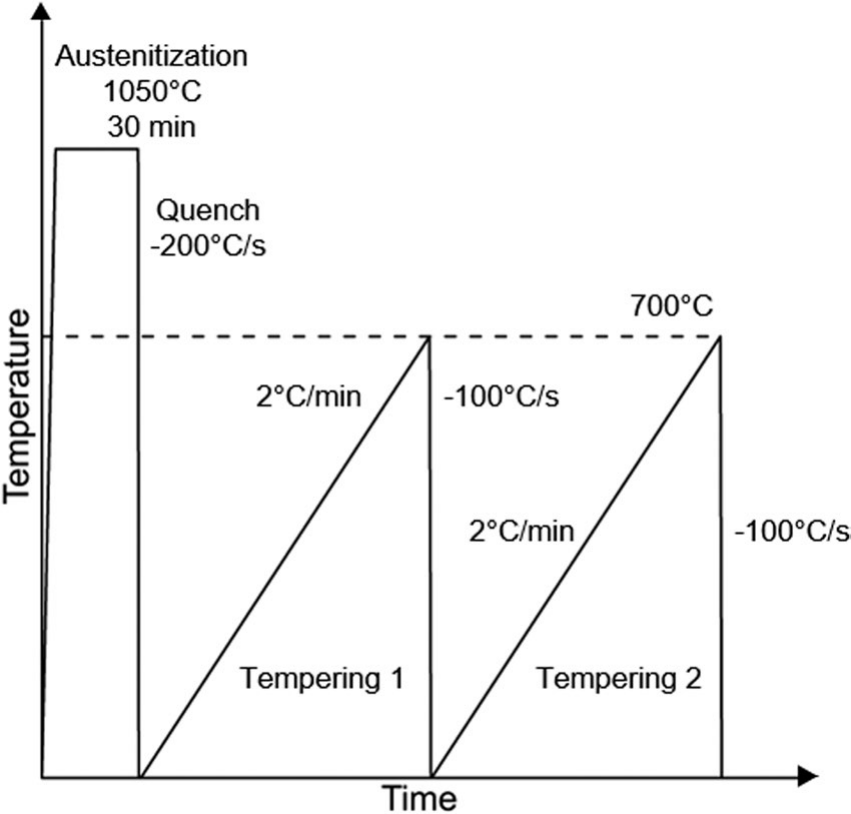
Schematic thermal profile utilized for the non-isothermal tempering analysis.

A Thermal Analysis (TA) Quenching dilatometer 805 L was utilized for non-isothermal analysis using cylindrical samples measuring 4 mm in diameter and 10 mm in length. Figure 1 shows the thermal profile employed for the non-isothermal procedure, hereafter labeled double tempering. The majority of the tempering reactions take place during the first tempering step, and even though equilibrium is not reached at the different temperatures, significantly less tempering reactions are expected during the second tempering step, and the latter is therefore used as a baseline for data analysis. Hence, subtraction of change in length observed in the second step from the first helps to highlight changes associated with the tempering process. A derivative of the difference in the change in length between first and second tempering steps with respect to temperature plotted versus temperature was utilized to evaluate the tempering response.

Based on the double tempering results, non-isothermal tempering was interrupted at various temperatures during the first tempering step and quenched to room temperature for further characterization. Samples utilized for hardness measurements and TEM analysis were produced through dilatometry whereas Mössbauer spectroscopy, XRD, and electrical resistivity specimens were heat treated in a box furnace. The heating rate of 2 °C/min was successfully simulated in the box furnace and cycles were interrupted using oil quenching.

Vickers micro-hardness samples were mechanically ground and subsequently polished using diamond suspensions of 6, 3 and 1 µm. Samples were then analyzed using the automated Vickers indenter LECO AMH55 applying a load of 0.98 N with a dwell time of 10 s. A total of 36 indents per sample was utilized.

Mössbauer spectra were acquired at room temperature with a ^57^Co-Rh source. Each sample was thinned down to 20–30 µm. Data were collected until the off-resonance baseline was 1–3 × 10^6^ counts per channel. The counts for each channel were normalized with respect to the total baseline counts and sub spectra were fitted using Lorentzian line shapes with WinNormos V3.0 coupled with IGOR Pro V6.3 software. Further details related to fitting and quantitative analysis of the various phases are described elsewhere^13,14^.

XRD specimens were mechanically ground to 1200 grit. Measurements were conducted with Cu-Kα radiation in a conventional θ/2θ diffractometer with a graphite monochromator on the detector side of the sample. Data were collected in a step-scan mode with a step size of 0.014° and time per step of 1.4 s. Peak broadening analysis utilizing the modified Williamson-Hall approach was used to determine dislocation density, which attributes all the strain responsible for peak broadening to dislocations assuming that contributions from internal stresses, texture, and other sources are negligible. This type of analysis is described in further detail elsewhere^15–17^.

A micro ohm meter Keysight 34420 A was used to perform electrical resistance measurements with the sample connected to Kelvin clips submerged in liquid nitrogen. Cylindrical samples measuring 1 mm in diameter and 50 mm in length were utilized. The method used consisted of four-wire resistance measurement applying a constant current of 10 mA.

**Figure 2:**
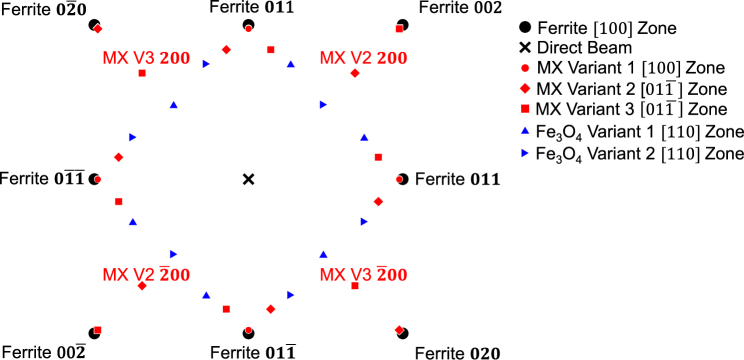
Schematic SADP of a [100] ferrite zone showing the three variants of the MX phase with the Baker-Nutting orientation relationship and two variants of Fe_3_O_4_. The SADPs of each phase were simulated using the JEMS© software package.

Transmission electron microscopy (TEM) was performed on selected conditions to evaluate MX (where M = Mo,V and X = C, N) precipitation that occurred at different non-isothermal tempering temperatures. Samples for TEM were taken from a metallographically prepared section of a dilatometry specimen. A focused ion beam (FIB) on an FEI Helios Nanolab 600i was used to prepare samples for TEM. Samples measuring 10 by 10 µm were thinned to less than 200 nm using a 30 keV gallium ion beam. Final preparation was conducted with a 2 keV gallium ion beam. An FEI Talos F200X TEM using a field emission gun operated at 200 keV was used for centered dark field (CDF) imaging. The CDF technique was used to evaluate the degree of precipitation of the MX phase which has a B2 rock salt prototype crystal structure. Figure 2 shows a schematic [100] ferrite selected area diffraction pattern (SADP) that includes SADPs from MX and Fe_3_O_4_ phases. The Baker-Nutting orientation relationship is often reported in the literature when MX forms in ferrite^18^, and was assumed for all CDF imaging. The $00\bar{2}$ ferrite **g** vector near the [100] ferrite zone was used for all bright field (BF) images in all cases. The 200 type MX **g** vector from the same two beam conditions used for BF images was used for CDF imaging. In order to evaluate TEM sample thickness, the convergent beam electron diffraction (CBED) technique using the $00\bar{2}$ ferrite **g** vector was used^19,20^. The reported precipitate size corresponds to the diameter of round precipitates and the long dimension of the elongated precipitates. At least 40 precipitates were measured in order to calculate the mean and 95 pct confidence limits of the precipitate sizes.

The datasets generated during and/or analyzed during the current study are available from the corresponding author on reasonable request.

## Results

### Dilatometry

The main difference between the non-isothermal double tempering approach and the conventional non-isothermal tempering utilized by several authors^5–9^ is the addition of a second non-isothermal tempering step to be utilized as a baseline. This approach more clearly reveals features associated with the different stages of tempering and reduces the impact on change in length caused by the temperature dependence of thermal expansion coefficient.

**Figure 3:**
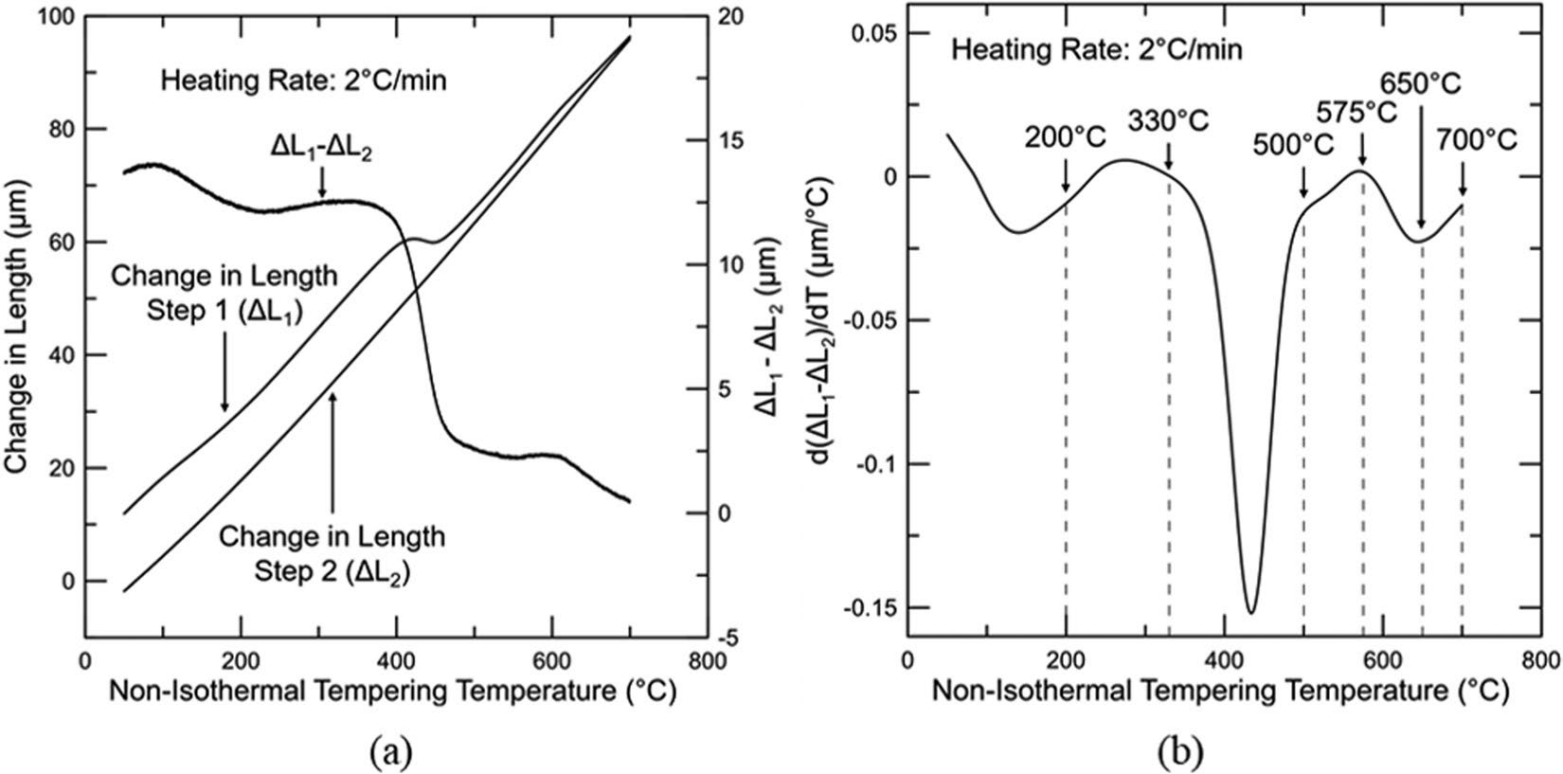
(**a**) Change in length of the first (ΔL_1_) and second (ΔL_2_) tempering steps and their difference versus temperature for a medium carbon steel. (**b**) Non-isothermal double tempering plot displaying the derivative of the difference between the change in length for the first and second tempering steps with respect to temperature (d(ΔL_1_–ΔL_2_)/dT) versus non-isothermal tempering temperature. Both tempering steps were conducted at a heating rate of 2 °C/min. Dotted lines indicate temperatures where tempering reactions of interest occur.

Figure 3(a) shows the change in length associated with the first and second steps of tempering, ΔL_1_ and ΔL_2_ respectively, as well as the difference in change in length from both steps (ΔL_1_-ΔL_2_). Each change in length was calculated utilizing the initial length (time = 0 s in the thermal profile shown in Fig. 1) as reference.

Figure 3(b) shows the derivative of the difference between the change in length for the first and second tempering steps with respect to temperature (d(ΔL_1_–ΔL_2_)/dT) versus non-isothermal tempering temperature. Several tempering reactions can be identified on the plot, which will be discussed in order of increasing temperature. The plot reveals a d(ΔL_1_–ΔL_2_)/dT trough from 50 to 200 °C, which is associated with the removal of carbon from solution for clustering and/or transition carbide formation^6,9^. At about 140 °C, a maximum rate of clustering/transition carbide precipitation is observed. At a temperature close to 200 °C, an increase in d(ΔL_1_–ΔL_2_)/dT indicates the start of the decomposition of austenite to ferrite causing an increase in length^7,21^. The temperature of 200 °C was chosen for analysis based on work by Bala *et al*.^10^. Cementite formation starts at around 300 °C and is characterized by the drop in d(ΔL_1_–ΔL_2_)/dT caused by the carbon diffusion out of martensite, which produces an overall reduction in the specific length and is detected in the non-isothermal approach as a reduction in the rate of change in length with temperature^22^. Even though the cementite formation is initiated at 300 °C, the temperature of 330 °C was chosen for analysis to capture a small level of cementite confirming precipitation start prior to this temperature. Stages II and III seem to overlap significantly^9^. The greatest rate of cementite precipitation is identified as a minimum in d(ΔL_1_–ΔL_2_)/dT at approximately 430 °C. The end of cementite formation takes place at around 500 °C and its dissolution needed to provide C for alloy carbide formation is subsequently initiated causing an increase in d(ΔL_1_–ΔL_2_)/dT due to the increase of carbon in solution, expanding the lattice^23^. Yamasaki *et al*.^24,25^ also reported cementite dissolution during tempering at elevated temperatures of ternary alloys of the types Fe-C-V and Fe-C-Mo. Additionally, Crafts *et al*.^11^ performed dilation analysis of secondary hardening in martensitic steels utilizing isothermal and non-isothermal procedures and observed an expansion due to the precipitation of Mo and V carbides. The curve reaches a peak at 575 °C followed by a decrease in d(ΔL_1_–ΔL_2_)/dT which may be related to segregation of Mn to cementite^26,27^. Mn segregation to cementite simultaneously decreases the ferrite lattice parameter^28^ and reduces the unit cell volume of cementite. Kagawa *et al*.^29^ measured the impact of Mn segregation to cementite utilizing XRD and detected a decrease in the unit cell volume of approximately 0.5 × 10^−3^ nm^3^ for cementite containing 4.85 wt pct Mn. Additionally, Ande *et al*.^30^ studied cementite with alloying element enrichment by Mn amongst others based on density functional theory (DFT) and reported a subtle decrease in the cementite unit cell volume associated with the presence of Mn on Fe sites. Finally, an increase in d(ΔL_1_–ΔL_2_)/dT is obtained between 650 and 700 °C. It is unclear whether the observed behavior is associated with a specific tempering reaction. Changes in this temperature range will be discussed based on the various techniques utilized.

### Vickers Micro-Hardness

**Figure 4:**
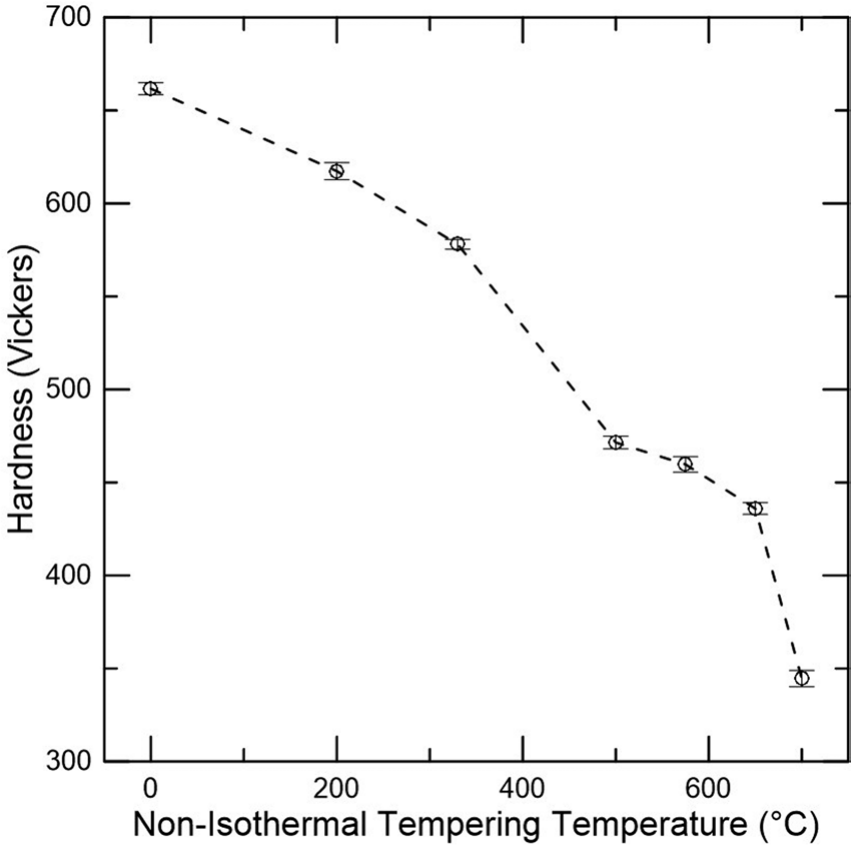
Hardness as a function of non-isothermal tempering temperature for a medium carbon steel. Specimens heated at a heating rate of 2 °C/min and quenched once the desired non-isothermal tempering was obtained. Error bars indicate 95 pct confidence limits. The point at the lowest temperature represents the as-quenched condition.

The tempering resistance of the studied alloy is demonstrated in Fig. 4 through the evolution in hardness. A progressive softening is observed with increased temperature up to 500 °C and secondary hardening is observed between 500 and 650 °C.

The softening between the as-quenched condition and 200 °C is likely associated with reduction in solid solution strengthening as carbon clustering and precipitation of transition carbides occurs. An increase of the tempering temperature up to 330 °C causes further decrease in hardness, which may be attributed to the start of retained austenite decomposition and cementite precipitation (stages II and III of tempering). A significant decrease in hardness is observed between 330 and 500 °C. This abrupt change in hardness is consistent with the cementite precipitation expected to take place within this temperature range based on dilatometry analysis (Fig. 3(b)) which removes the solid solution strengthening effect associated with carbon in the martensitic matrix^31^. Softening is decreased between 500 and 650 °C, indicating that alloy carbide precipitation occurs (stage IV of tempering), hardening the material and possibly delaying the recovery process. Finally, a significant decrease in hardness is obtained at 700 °C suggesting substantial coarsening of precipitates, which allows for a greater extent of recovery and possibly recrystallization to occur.

### Mössbauer Spectroscopy

Mössbauer spectroscopy was conducted to assess and quantify the levels of transition carbides, cementite and retained austenite. The small amounts of carbides in medium carbon steels requires a careful method to detect and quantify them in the presence of the dominant martensite/ferrite resonance signal. Such a method was recently developed and presented in detail by Pierce *et al*.^13,14^. Through careful fitting of the spectra via monitoring of the χ^2^-fitting parameter, small amounts of magnetic carbides well below 0.5 pct resonance area could be found based on their contribution to the spectra in velocity regions where the martensite/ferrite resonance was minimal^13,14^. The presence of the η-transition carbide was confirmed by TEM^13,14^. Cementite was also confirmed by TEM for a more elevated temperature treatment^14^. The alloy studied here has C, Mn, and Si contents quite similar to those of the alloy used by Pierce *et al*.^13,14^ so that same methodology was applied here to quantify the distribution of C in the carbides and the retained austenite for the different processing temperatures.

**Figure 5:**
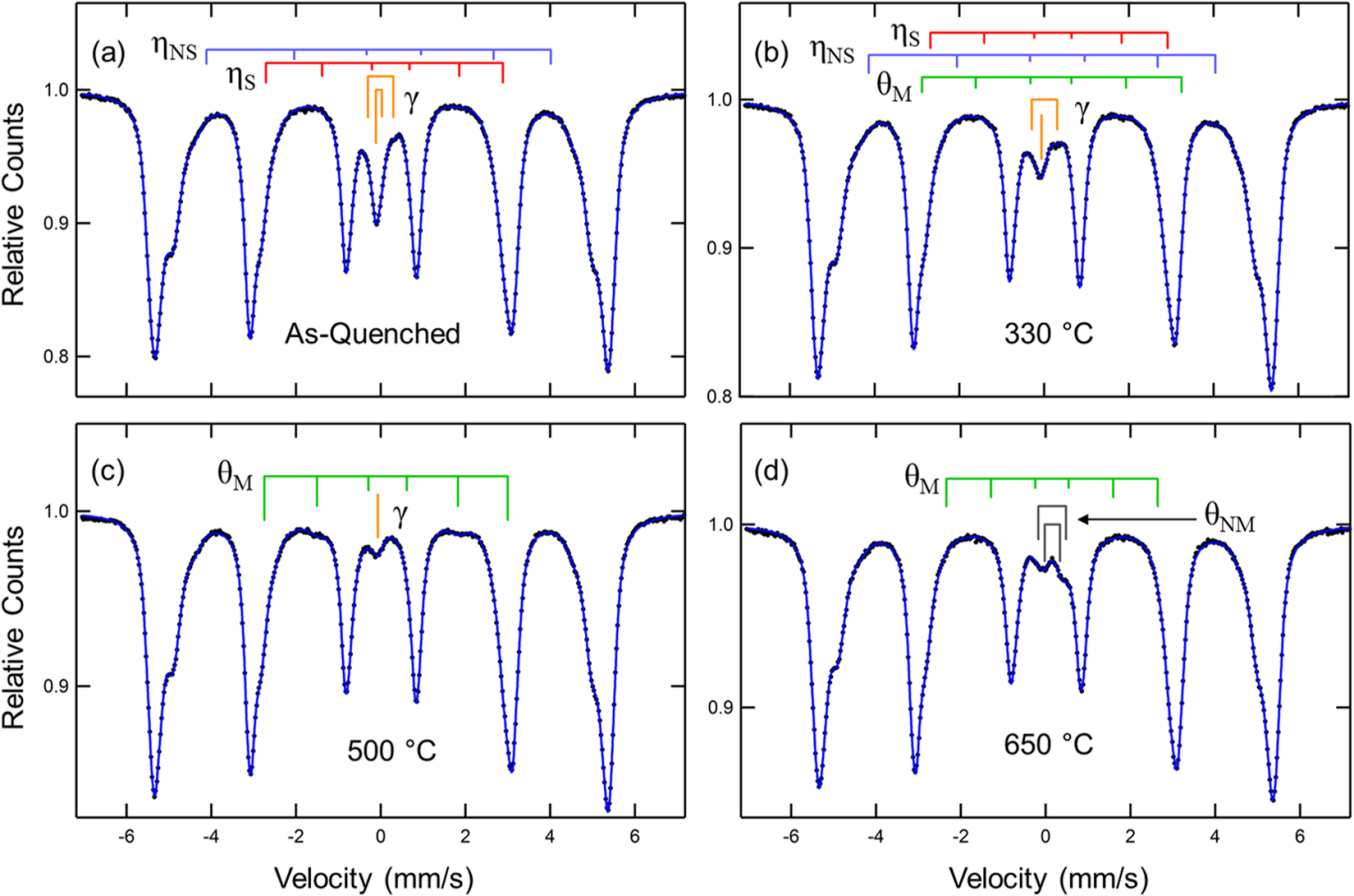
Mössbauer spectra for the (**a**) as-quenched martensite and samples non-isothermally tempered to (**b**) 330 °C, (**c**) 500 °C, and (**d**) 650 °C for a medium carbon steel. The stick diagrams represent the resonance attributed to stoichiometric η carbide (η_S_), non-stoichiometric η carbide (η_NS_), retained austenite (γ), magnetic cementite (θ_M_), and non-magnetic cementite (θ_NM_). The stick diagrams are not to scale and components associated with martensite and ferrite have been removed for better visualization. The solid line through the data points is the fitted curve.

Figure 5 shows the Mössbauer spectra and fits for the as-quenched condition and for samples non-isothermally tempered to 330, 500 and 650 °C to display the significant changes found. Each spectrum was fitted with multiple subspectra until an optimum χ^2^ was found. Combinations of magnetic sextets (with parameters of magnetic hyperfine field, B_hf_, isomer shift, IS (relative to the center of the pure Fe calibration), quadrupole splitting, QS, line width, W, and resonance area, A), non-magnetic doublets (with parameters IS, QS,W and A) and single lines (with parameters IS,W and A). The stick diagrams label the carbides identified (stoichiometric Fe_2_C-like carbide, η_S_, non-stoichiometric Fe_3_C-like carbide, η_NS_, magnetic cementite, θ_M_, and non-magnetic cementite θ_NM_), as well as the retained austenite, γ. The major part of the resonance (no stick diagrams shown) is the martensite/ferrite (α) resonance and required multiple sextets due to the distribution of local environments produced by the alloy additions. This phenomenon is well known^32,33^. Due to sample thickness effects, these multiple spectra can also account for distortions in the Lorentzian line shape. The contributions to the α resonance are used later to demonstrate depletion of Mn from the α phase and support the identification of the θ_NM_.

**Figure 6:**
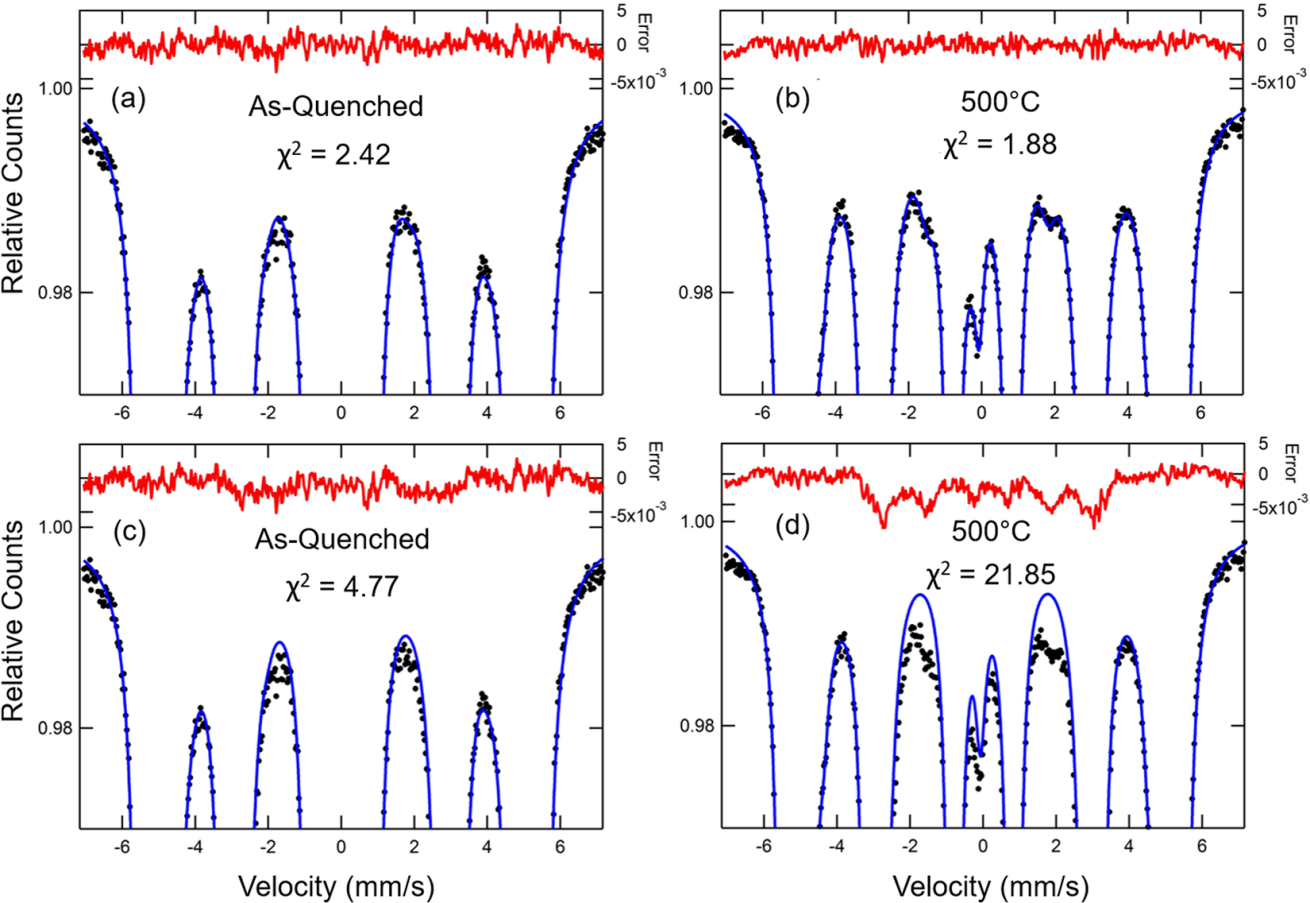
Mössbauer spectra with the optimized fit and error signals on an expanded vertical scale for (**a**) as-quenched martensite and (**b**) sample non-isothermally tempered to 500 °C; and Mössbauer spectra with the non-optimized fit and error signals on an expanded vertical scale for (**c**) as-quenched martensite without the subspectra for η_S_ and η_NS_ and (**d**) sample non-isothermally tempered to 500 °C without the subspectra for η_S_, η_NS_ and θ_M_. The fitting parameter χ^2^ is provided in each plot and demonstrates the improvement in the fit with additions of subspectra for η_S_, η_NS_ and θ_M_ phases. The black dots represent the experimental data whereas the blue solid lines are associated with the fit. Red lines on the upper part of the plots represent the difference between fit and experimental data. Results represent analysis performed with a medium carbon steel.

The retained austenite (γ) resonance is easily detected due to its non-magnetic nature near the center of the velocity range, well away from the inner lines for the α resonance. The subspectra associated with γ are well known to be associated with Fe in various C environments with established IS and QS values^13^. The Fe with one C nearest neighbor is partially resolved as a quadrupole doublet. With a good fit to α and γ resonances, the presence of the weak carbide signals can be sought starting with spectral parameters found in previous studies of transition carbides and cementite. The detection is made via a significant improvement in the χ^2^ as illustrated in Fig. 6. Figure 6(a) and (b) show the as-quenched and 500 °C sample spectra, respectively, with the optimized fit along with the error signals (difference between fit and experimental data). The as-quenched spectrum fit includes η_S_ and η_NS_ components (shown by the stick diagrams in Fig. 5(a)). The 500 °C spectrum fit includes θ_M_ indicated by the stick diagram in Fig. 5(b). Figure 6(c) and (d) show the fits on an expanded vertical scale without the η_S_ and θ_M_ included in the fits where clear differences in the fit and data can be seen near −2 mm/s and + 2 mm/s, and the error signal indicates magnetic components. For the as-quenched sample, the χ^2^ value is then more than a factor of 2 larger. The area of the magnetic component is found to be 1.4 pct and has spectral parameters as follows: B_hf_ = 17.3 ± 0.4 T, IS = 0.15 ± 0.06 mm/s, QS = −0.2 ± 0.1 mm/s in good agreement with the values documented for η_S_^13^. If the η_NS_ component (0.8 pct area) is left out of the fit, the χ^2^ value increases from 2.42 to 3.22, a significant increase. The η_NS_ is detected primarily via its larger B_hf_ = 25.0 ± 0.5 T, again in agreement with the value documented^13^. The presented spectral parameters associated with transition carbides reported here display general agreement with results found in literature for η- and ε-carbides, which show similar parameters^34–36^. TEM confirmation of the presence of η-carbide in an alloy with similar carbon levels and heat treatment^13^ indicates that the subspectra associated with transition carbides must be attributed to η-carbide in the present work. For the 500 °C sample, the χ^2^ value increases by more than a factor of 10. The area of the magnetic component is 5.0 pct and has spectral parameters as follows: B_hf_ = 17.8 ± 0.2 T, IS = 0.14 ± 0.02 mm/s, QS = −0.03 ± 0.03 mm/s. The IS and QS are similar to literature values for cementite^37^ but the B_hf_ value is significantly lower than that of pure cementite (20.7 T) and this is attributed to the incorporation of the available alloying elements shown in Table 1. The effect of Mn on B_hf_ in (Fe_1−x_Mn_x_)_3_C cementite has been documented by Schaaf *et al*.^38^ for x up to 0.15. For a Mn content x = 0.06, the B_hf_ decreases to about 16 T, while at x = 0.15, the B_hf_ = 0 T and the material is non-magnetic. Finally, the line widths suggested by the error signals are quite broad for the outer lines of the magnetic sextets. This broadness was considered in the fitting with line width ratios W1/W3 = 1.6 and W2/W3 = 1.3, where the six lines of the sextet are numbered 1–6 from negative to positive velocity, and they are assumed to be pairwise equal, *i*.*e*. W1 = W6, W2 = W5, W3 = W4. Typical values for W3 are 0.4 ± 0.1 mm/s. The relative areas of the six lines of the carbide sextets were fixed at 3:2:1:1:2:3 as appropriate for random orientations of the magnetic fields.

Similarly, analysis of the other spectra in Fig. 5 via careful monitoring of the χ^2^ values show the following trends suggested by the stick diagrams: at 330 °C, the amount of γ is significantly reduced and θ_M_ is detected (0.5 pct area); at 500 °C, γ is further reduced, no η-carbides are detected and θ_M_ has increased to 5 pct area; at 650 °C, no γ is found and a significant new quadrupole doublet appears that can be attributed to Mn-enriched cementite, θ_NM_. The latter is best fit by two quadrupole doublets with IS and QS values that agree with those found by Schaaf *et al*.^38^ for (Fe_0.85_Mn_0.15_)_3_C. These correspond to the two crystallographic Fe sites in cementite, Fe_a_ and Fe_b_^38^. The B_hf_ of the θ_M_ decreases systematically with increasing temperature above 330 °C consistent with increasing Mn enrichment of the magnetic phase (19, 17.8, 17.1, 15.4, and 14 T at 330, 500, 575, 650, and 700 °C, respectively). Based on a plot of B_hf_ versus Mn in cementite^38^ one can estimate the quantitative enrichment of Mn.

All the areas found for γ, η_S_, η_NS_, θ_M_, θ_NM_ are then converted to Fe atomic fractions by first making sample thickness corrections according to the method described in detail elsewhere^13^. These corrections typically reduce area by as much as 15–25 pct for 20–30 µm sample thicknesses and are therefore important corrections. Then recoilless fraction, f, corrections are made based on the following values: f(α) = 0.82, f(γ) = 0.81, f(η) = 0.89 (no difference assumed for η_S_ and η_NS_)^13^ and f(θ) = 0.86 (no difference is assumed for θ_M_ and θ_NM_)^14^. Also, all Fe sites within each phase are assumed to have the same f-value. Note that these corrections amount to 9 pct or less.

**Figure 7:**
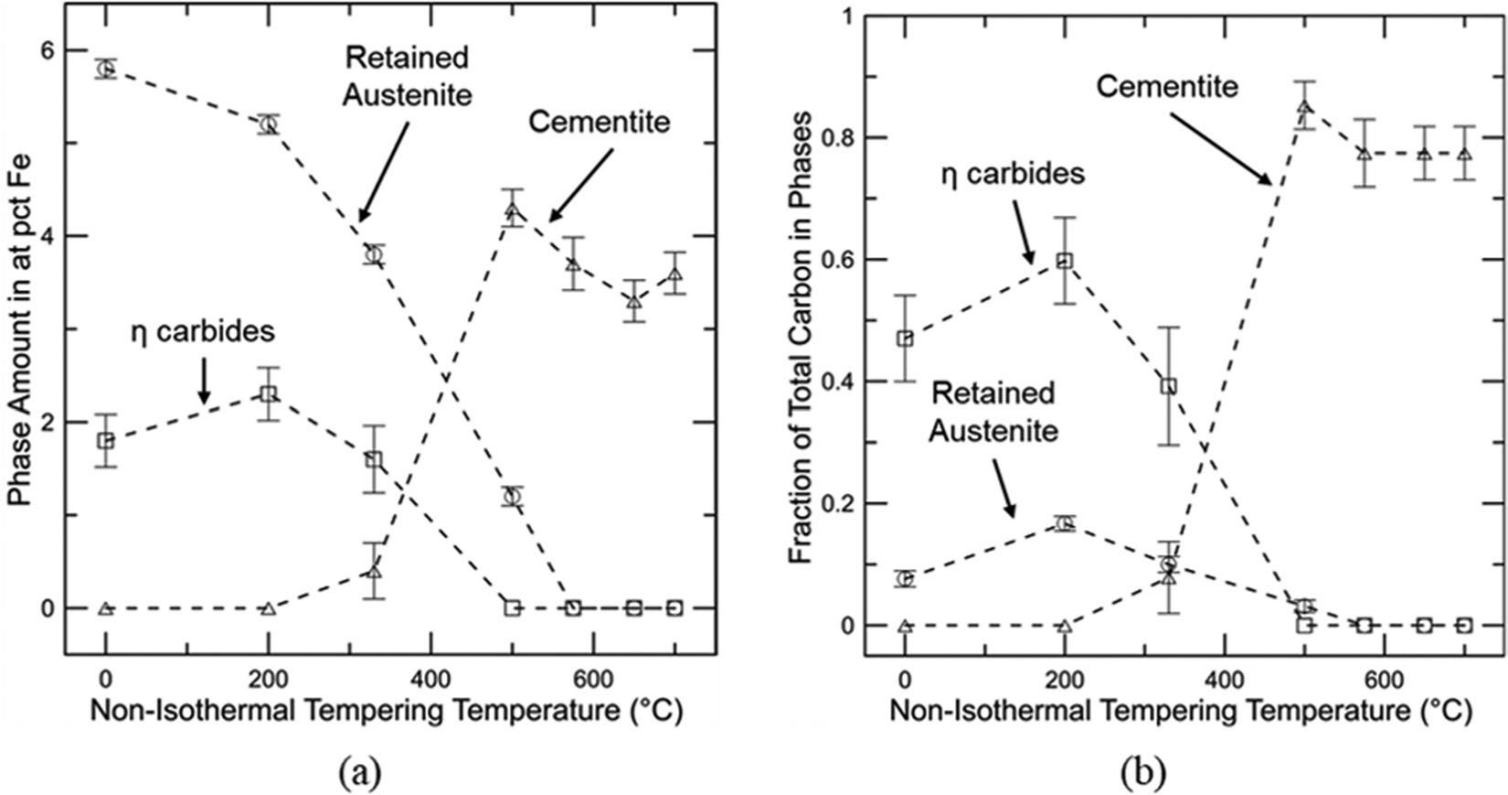
(**a**) Quantitative evaluation of η carbides, cementite, and retained austenite performed with Mössbauer spectroscopy for a medium carbon steel submitted to non-isothermal tempering up to different temperatures (**b**) Fraction of total bulk carbon in η carbides, cementite and retained austenite for the different non-isothermal tempering temperatures. Error bars indicate the total uncertainty associated with the data fitting and reproducibility.

Figure 7(a) presents a compilation of Mössbauer spectroscopy results for the as-quenched material and specimens non-isothermally tempered to 200, 330, 500, 575, 650, and 700 °C with a quantitative evaluation of η carbides, cementite, and retained austenite. Results obtained for η_S_ and η_NS_, and θ_M_ and θ_NM_ were summed and labeled as η carbide and cementite, respectively. The presence of the transition carbide in the as-quenched sample demonstrates significant auto-tempering during the quenching step. The amount of η carbide does not change significantly up to 200 °C followed by a slight decrease at 330 °C, and they are not observed at higher tempering temperatures as they are possible nucleation sites for cementite precipitation. The amount of retained austenite decreases slightly from as-quenched to 200 °C with a more substantial drop for tempering temperatures higher than 200 °C, suggesting that stage II is initiated at around 200 °C. Cementite is initially detected at 330 °C. Between 330 and 500 °C, the amount of cementite precipitation increases. This temperature range is associated with stage III. The overlap between stages II and III is clear. For tempering temperatures of 500 °C and higher, the amount of Fe in cementite decreases due to Mn segregation to cementite, *i*.*e*. the Fe fraction in the (Fe_1−x_Mnx)_3_C phase, as detected by the ^57^Fe Mössbauer signal, goes down as x increases, even though the amount of cementite may remain constant or decrease with increasing tempering temperature. The simultaneous presence of θ_M_ and θ_NM_ is first detected at 575 °C with 2.7 ± 0.2 and 1.0 ± 0.2 at pct Fe of θ_M_ and θ_NM_, respectively. However, at 650 °C, the amount of θ_M_ becomes smaller than θ_NM_, 0.5 ± 0.2 and 2.8 ± 0.10 at pct Fe, respectively, showing continued enrichment of Mn in cementite as the tempering temperature increases.

Figure 7(b) shows the fraction of the total bulk carbon present in retained austenite, η carbides and cementite. The carbon amounts (C) in the Fe-containing carbides were calculated based on the Fe fractions (F) determined by Mössbauer spectroscopy (Fig. 7(a)), stoichiometry (Y), and the atomic fraction of substitutional alloying elements estimated in each phase (a):

1 $${\rm{C}}=\frac{{\rm{F}}\,}{{\rm{Y}}(1-{\rm{a}})}$$

For η_S_, Y = 2, whereas for η_NS_, θ_M_ and θ_NM_, Y = 3. The fraction of substitutional alloying elements was dependent on assumptions for the different conditions. For the as-quenched, 200 and 330 °C conditions, para-equilibrium transformations were assumed and thus a = 0.059. For the condition tempered to 500 °C, a(θ_M_) = 0.059 + x(Mn), where x(Mn) is the extra Mn enrichment based on B_hf_ (θ_M_). The a(θ_M_) for the conditions tempered from 575 to 700 °C was calculated as a(θ_M_) = 0.055 + x_Mn_, which assumes loss of Mo and V to alloy carbides. Finally, for θ_NM_, which is present in the samples tempered from 575 to 700 °C, a(θ_NM_) = 0.055 + x_Mn_, where the extra Mn enrichment is determined to account for all of the carbon.

The carbon content in retained austenite was determined based on the fraction of Fe sites in austenite with no carbon atom nearest neighbors^14^:

2 $${\rm{r}}(0)={(1-\frac{{{\rm{X}}}_{{\rm{C}}}}{1-{{\rm{X}}}_{{\rm{C}}}})}^{6}$$

where X_C_ is the atomic fraction of carbon in austenite. Equation (2) assumes a random distribution of C in austenite^34^. From the as-quenched condition to 200 °C, no variation outside of the range of uncertainty in carbon levels of η carbides is observed whereas retained austenite displays an increase in the C level due to C partitioning. At 330 °C, the amount of carbon in η carbides and retained austenite decreases. A low fraction of carbon in cementite is detected at 330 °C, which is consistent with the loss of retained austenite and η carbides for stages II and III of tempering. Raising the temperature to 500 °C causes a large increase in the carbon fraction in cementite related to stage III of tempering. At this temperature, carbon is no longer observed in η carbides and only a small amount is found in retained austenite. A further increase in temperature does impact the carbon levels in cementite. Comparison between carbon levels in cementite at 500 and 650 °C suggests cementite dissolution taking place in this temperature range which is consistent with results found by Ustinovshchikov^23^ for 0.23C–2.65Mo and 0.17C–1.43 V wt pct steels.

Mössbauer spectroscopy is only able to directly evaluate Fe-containing phases and thus assessment of alloy carbides is not possible utilizing this technique. However, the presence of substitutional elements in solution in martensite and ferrite can affect the spectral parameters. Consider the resonance-area-weighted average magnetic field, <B_hf_(α)>, calculated from the multiple component fits to the martensite/ferrite (α) resonance. Nickel has been reported to increase the <B_hf_(α)> while Si, Mn, V, Mo, and Al are known to reduce it^32,33^. Using the results from Cadeville *et al*.^39^ for pure Fe-C martensite, one can show that <B_hf_(α)> is not changed by the presence of C due to cancelling effects of the two nearest neighbor sites. Thus, monitoring <B_hf_(α)> versus tempering temperature may provide evidence for alloying element partitioning. In addition, there are partially resolved components in the α resonance that can be seen in Fig. 5. The obvious shoulders on the outer lines of the sextet at ±4.9 mm/s (B_hf_(α) = 30.6 T) correspond to a component primarily due to Fe atoms with one non-magnetic alloying element nearest or next nearest neighbor^32,33^, with an Fe fraction designated α(1); weaker shoulders at ±4.4 mm/s (B_hf_(α) = 27.6 T), correspond primarily to Fe atoms with two non-magnetic alloying elements nearest neighbor^32,33^, with a much smaller fraction. The latter component may also contain a contribution from C in solution in the martensite based on the results of Cadeville *et al*.^39^. which show the component due to 1 nearest neighbor of C along the c-axis of the body-centered-tetragonal unit cell with B_hf_(α) = 26.9 T, similar to the Fe sites with 2 nearest neighbors of non-magnetic alloying elements. This component is designated α(2 + C). Thus, the presence of significant carbon in solution in martensite may be detectable via α(2 + C). However, C clustering along dislocations or other defects are not directly detectable due to the small Fe fraction affected.

Table 2 shows <B_hf_(α)>, α(1) and α(2 + C). Errors provided indicate the total uncertainty associated with data fitting and reproducibility. The lack of change in <B_hf_(α)> up to 330 °C is consistent with no alloying element partitioning as expected based on the high activation energy for diffusion of substitutional elements. A slight loss of alloying elements appears to take place between 330 and 500 °C due to the small increase in <B_hf_(α)>. From 500 to 650 °C, the increase.of <B_hf_(α)> indicates the loss of alloying elements from solution in ferrite possibly associated with alloy carbide precipitation and segregation to cementite. Between 650 and 700 °C no change in <B_hf_(α)> is detected. The component α(1) presents no obvious change up to 500 °C and then it clearly drops for the higher temperatures, which is consistent with a loss of some fraction of the alloying elements from solution. For temperatures higher than 575 °C, α(1) remains constant and hence no evidence of partitioning is observed with this parameter. Finally, the α(2 + C) shows a significant drop from the as-quenched condition up to 330 °C whereas <B_hf_(α)> and α(1) do not change, consistent with some carbon loss from solution in martensite. Since not much diffusion is expected for substitutional elements up to 500 °C, the decrease in α(2 + C) between 330 and 500 °C can also be associated with the loss of carbon from martensite, which is consistent with the significant precipitation of cementite in this temperature range. The further drop in this component must be due to loss of substitutional alloying elements from solution. Interestingly, no variation between 650 and 700 °C is observed for the α(2 + C) component, similar to <B_hf_(α)>.

**Table 2:** Martensite/Ferrite Spectral Parameters versus Heat Treatment.

Condition	<B_hf_(α)> (T)	α(1)	α(2 + C)
As-Quenched	31.88 ± 0.01	0.40 ± 0.01	0.037 ± 0.002
200 °C	31.87 ± 0.01	0.41 ± 0.02	0.034 ± 0.003
330 °C	31.89 ± 0.01	0.41 ± 0.01	0.031 ± 0.003
500 °C	31.94 ± 0.01	0.40 ± 0.01	0.025 ± 0.002
575 °C	32.05 ± 0.01	0.35 ± 0.01	0.022 ± 0.002
650 °C	32.08 ± 0.01	0.36 ± 0.01	0.015 ± 0.002
700 °C	32.08 ± 0.01	0.34 ± 0.01	0.016 ± 0.002

### Electrical Resistivity and X-Ray Diffraction

Electrical resistivity is a material property measuring how effective a material resists carrying an electrical current. Based on Matthiessen’s rule, Equation (3) describes the total resistivity of a specimen (ρ) as the sum of the individual contributions from thermal vibrations (ρ_T_), residuals (ρ_R_), and dislocations (ρ_D_)^40^.

3 $${\rm{\rho }}={{\rm{\rho }}}_{{\rm{T}}}+{{\rm{\rho }}}_{{\rm{R}}}+{{\rm{\rho }}}_{{\rm{D}}}$$

The residual resistivity, described in Equation (4). can be subdivided into the resistivity due to carbon (ρ_C_) and the resistivity associated with other elements (ρ_I_) such as Mn, Si, Ni, Mo, V, and Al^41,42^. An increase in any of the aforementioned variables promotes an increase in the total resistivity due to greater electron scattering:

4 $${{\rm{\rho }}}_{{\rm{R}}}={{\rm{\rho }}}_{{\rm{C}}}+{{\rm{\rho }}}_{{\rm{I}}}$$

**Figure 8:**
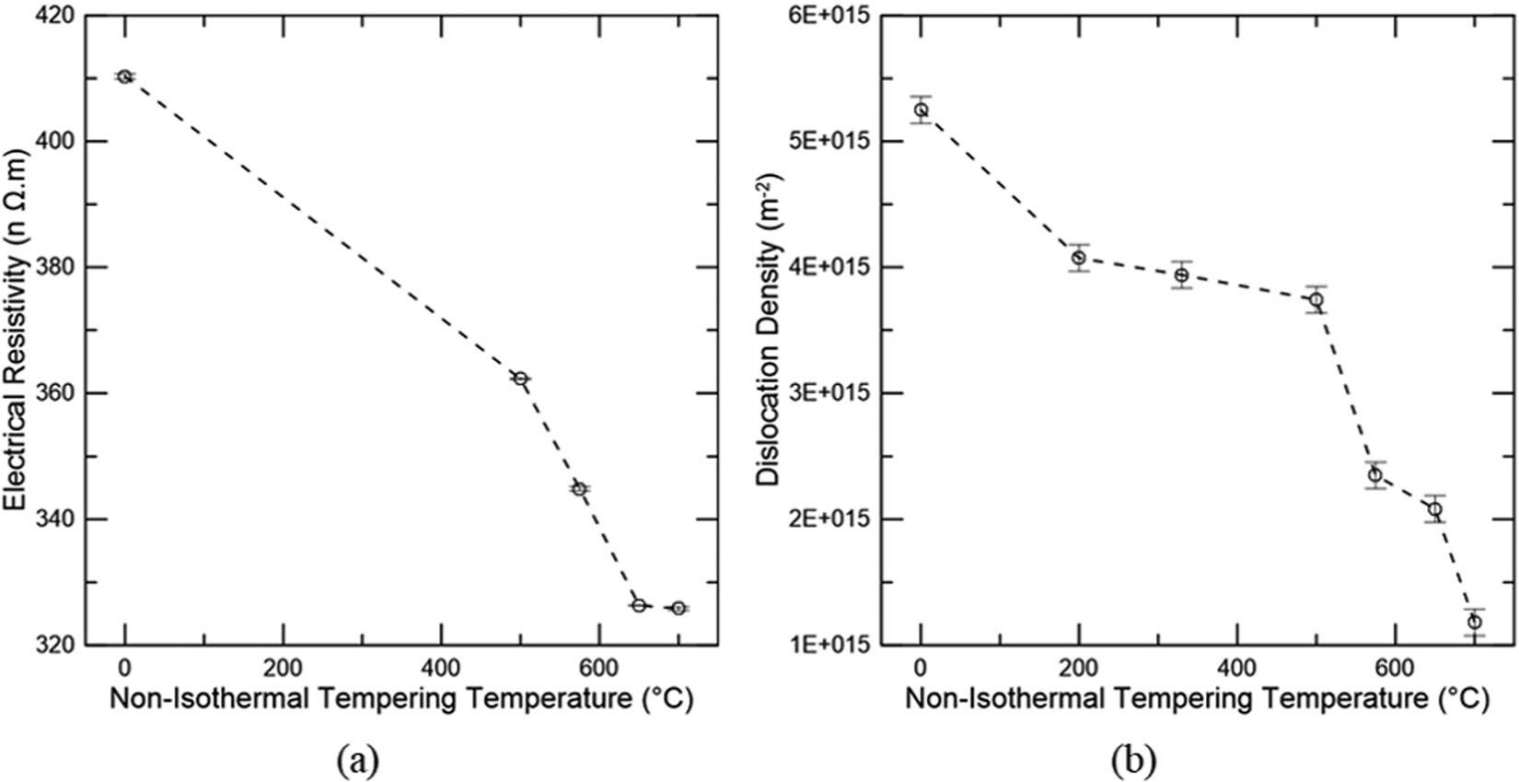
(**a**) Electrical resistivity and (**b**) Dislocation density for various non-isothermal tempering temperatures for a medium carbon steel. Error bars in (**a**) indicate the 95 pct confidence limits and (**b**) indicate the 95 pct confidence limits associated with three measurements performed for the as-quenched sample and assumed as the technique uncertainty.

Figure 8(a) shows electrical resistivity measurements versus non-isothermal tempering temperature while Fig. 8(b) displays the dislocation density obtained through analysis of peak broadening of the ferrite/martensite peaks from XRD analysis using the modified Williamson-Hall approach^15–17^. Between the as-quenched and the 500 °C measurements, no significant segregation of substitutional elements and precipitation of alloy carbides is expected. Hence, in this temperature range, no important variation in ρ_I_ should take place. In addition, since all measurements were performed in liquid nitrogen, ρ_T_ contributes insignificantly to the total resistivity. Therefore, the change in resistivity between as-quenched and 500 °C is only related to ρ_C_ and ρ_D_. The substantial reduction in resistivity in this temperature range is mainly attributed to the removal of carbon from solution for cementite precipitation. While Fig. 8(b) indicates there is a drop in dislocation density between the as-quenched and 200 °C, this decrease can at least partially be attributed to a decrease in tetragonality imposed to the martensitic crystal structure by carbon atoms upon tempering. Tetragonality in the as-quenched condition is associated with anisotropic distortion, which causes an apparent broadening to BCC/BCT diffraction peaks; peak broadening is used to calculate dislocation density in the Williamson-Hall analysis. However, the {222} peak did show a decrease in width at this temperature range although this peak is insensitive to the effects of tetragonality, suggesting there could be some dislocation recovery at lower tempering temperatures. With increased tempering temperature, carbon segregates to dislocations and the tetragonality effect is no longer observed. Thus, from 200 to 500 °C, a much smaller change in dislocation density is detected. The decrease in dislocation density is greater at high non-isothermal tempering temperatures, consistent with the general observation that recovery is significant above 500 °C.

At temperatures higher than 500 °C, ρ_I_ plays a significant role as alloy carbides form and should be additionally considered in the resistivity measurements. From 500 to 650 °C, resistivity (Fig. 8(a)) drops at an approximately constant rate whereas a significant decrease in dislocation density (Fig. 8(b)) is measured between 500 and 575 °C, and a smaller change is detected between 575 and 650 °C. These results suggest a significant contribution from recovery to the drop in resistivity from 500 to 575 °C. At higher temperatures between 575 and 650 °C, the recovery process is slowed down likely related to carbides pinning dislocations which may be due to a greater extent of alloy carbide precipitation at around 575 °C. Thus, the drop in resistivity from 575 to 650 °C is likely impacted by alloy carbide precipitation and Mn segregation to cementite. Finally, resistivity is unchanged between 650 and 700 °C, while a decrease in dislocation density is observed. Coarsening of MX likely takes place allowing for further recovery and perhaps some extent of recrystallization, producing the overall decrease in dislocation density. Thus, since resistivity is constant in this range of temperatures, an increase in ρ_R_ should be expected. However, the Mössbauer parameter <B_hf_(α)>, sensitive to alloy segregation, showed no change within the same temperature range.

**Figure 9:**
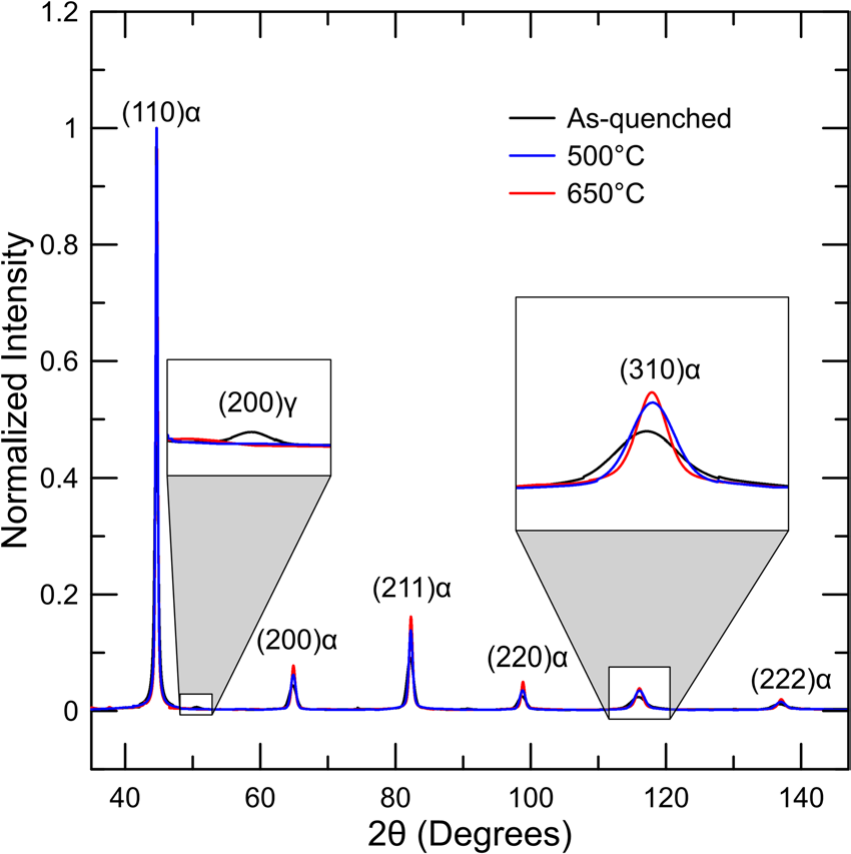
XRD patterns of as-quenched specimen and samples non-isothermally tempered to 500 and 650 °C for a medium carbon steel.

Figure 9 shows XRD patterns of the as-quenched specimen and samples non-isothermally tempered to 500 and 650 °C. Peaks associated with martensite/ferrite were indexed for the three, different conditions whereas an austenite peak was only indexed for the as-quenched sample which presented a significantly larger amount of this phase than the samples heat treated to 500 and 650 °C according to Mössbauer results (Fig. 7(b)). Peak broadening was clearly reduced with increased tempering temperatures as highlighted for the martensite/ferrite (310) peak. Lattice parameter evaluations of ferrite/martensite for the various conditions may facilitate analysis to detect removal and return of carbon and perhaps alloying elements to solution. Such analysis was performed, but no significant changes were observed between tempering temperatures, especially for high tempering temperatures, as shown in Fig. 9 by the significant overlap of the XRD patterns. The use of neutron diffraction and synchrotron X-rays may be helpful for this type of analysis due to the higher resolution and accuracy for peak position determination provided by these techniques.

### Transmission Electron Microscopy

**Figure 10:**
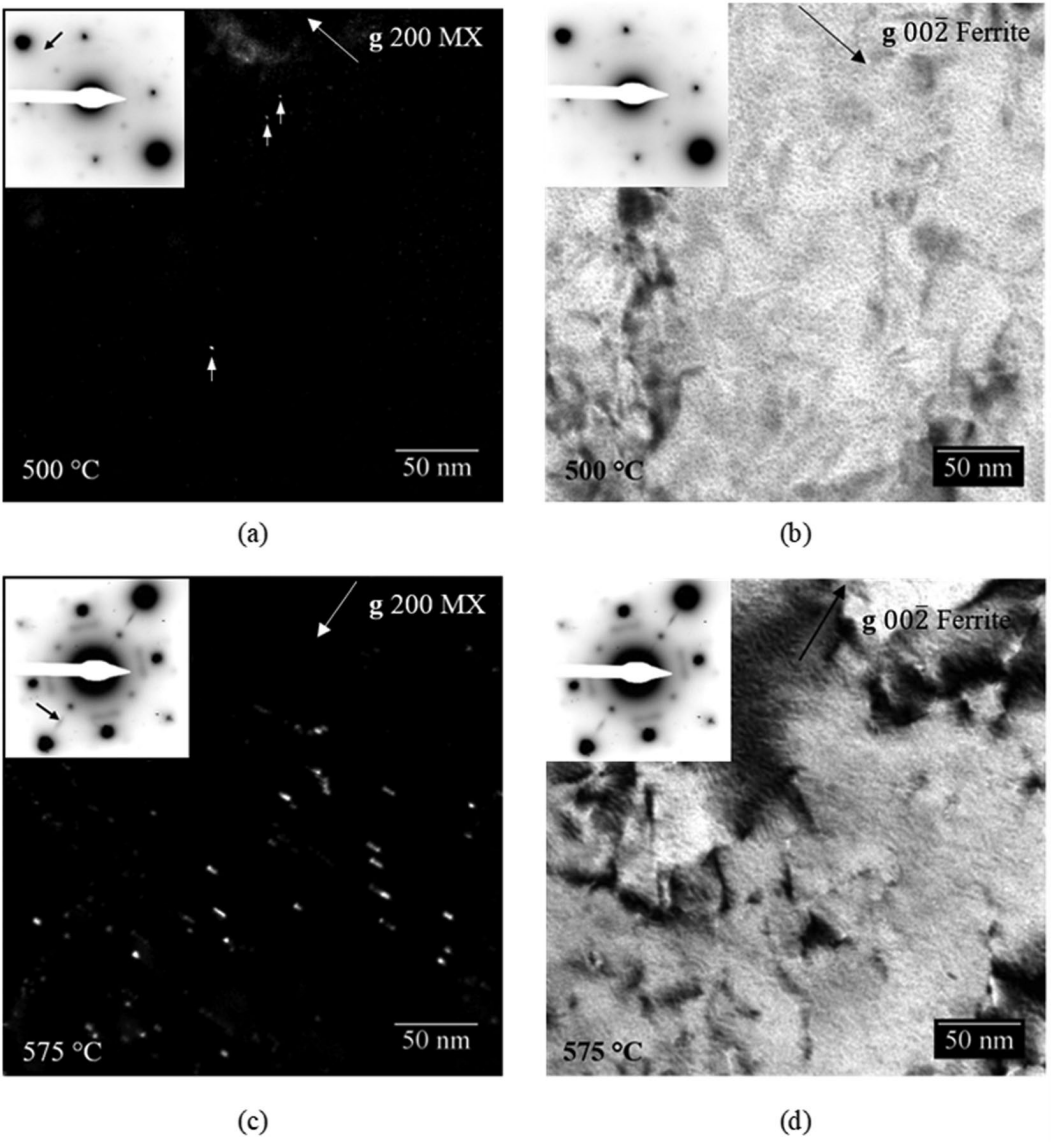
(**a**) TEM CDF micrograph of the sample non-isothermally tempered to 500 °C. (**b**) TEM BF micrograph of a sample non-isothermally tempered to 500 °C. (**c**) TEM CDF micrograph of the sample non-isothermally tempered to 575 °C (**d**) TEM BF micrograph of the sample non-isothermally tempered to 575 °C. All samples consist of a medium carbon steel. A heating rate of 2 °C/min was utilized for both samples. Selected area diffraction patterns (SADPs) for each micrograph are shown in the inset. Arrows in the SADPs of the CDF micrographs indicate the location of the objective aperture during CDF imaging. Arrows in the upper right corners indicate the orientations of the $00\bar{2}$ ferrite or 200 MX g vectors for BF and CDF micrographs, respectively.

TEM was performed on samples non-isothermally tempered to 500 and 575 °C in order to confirm that the precipitation of MX phases occurs within this temperature range where d(ΔL_1_–ΔL_2_)/dT is observed to increase in dilatometry data. Figure 10(a) shows a CDF micrograph of the 500 °C tempered condition and a corresponding bright field image is shown in Fig. 10(b). Very few precipitates can be observed in the micrograph. Arrows point to the sphere-shaped MX precipitates that can be observed. The mean size of the MX precipitates in the 500 °C tempered condition is 1.2 ± 0.7 nm. A significantly greater number of larger precipitates is observed after tempering at 575 °C as shown by Fig. 10(c). The precipitates in the 575 °C tempered condition appear to be elongated perpendicular to the 200 MX g vector indicated in the micrograph. The mean size of the MX precipitates in the 575 °C tempered condition is 5.3 ± 0.7 nm. The thicknesses of the TEM samples for the 500 and 575 °C tempered conditions are 132 ± 10 nm and 148 ± 10 nm, respectively. Considering that the sample thicknesses are comparable, and the number and size of the MX precipitates are much greater in the 575 °C tempered condition, it can be assumed that the volume fraction of the MX precipitates is significantly greater in the 575 °C condition compared to 500 °C, although further analysis of a larger number of specimens would be necessary to make a quantitative evaluation. For reference, BF pairs to the CDF micrographs are shown in Fig. 10(b) and (d) for the 500 and 575 °C tempered samples respectively. In summary, TEM CDF results show that a significant increase in the volume fraction of MX precipitates correlates with the increase in d(ΔL_1_–ΔL_2_)/dT between 500 and 575 °C observed in dilatometry.

## Discussion

The interpretation of microstructural evolution from the dilatometry double tempering approach was mostly corroborated by the other characterization techniques, which also made further interpretation possible.

At lower tempering temperatures, a trough is shown in Fig. 3(b) between 50 and 200 °C. Mössbauer spectroscopy revealed that no further precipitation of transition carbides was obtained with non-isothermal tempering to 200 °C and therefore this dilatometry signal was likely associated with segregation and clustering of carbon atoms as shown by others^7^. The observed presence of transition carbides in these conditions is then explained by auto-tempering during the quenching step prior to tempering. An increase in temperature to 330 °C caused an initial increase in the dilatometry derivative measurement followed by a slight decrease, which are attributed to retained austenite decomposition and start of cementite precipitation, respectively, based on Mössbauer spectroscopy analysis (Fig. 7(a)). Such analysis confirms the start of cementite precipitation during retained austenite decomposition. Overall, stages II and III were clearly captured by the dilatometric measurements, which was confirmed by the complementary technique Mössbauer spectroscopy. Finally, the trough situated between 330 and 500 °C in Fig. 3(b) implies cementite precipitation or stage III. The end of cementite precipitation was confirmed based on corroboration with Mössbauer spectroscopy, which showed a maximum amount of carbon in this phase at 500 °C. Hardness measurements were consistent with the provided analysis. The carbon removal from martensite for clustering and carbide precipitation reduces the solid solution strengthening component from the as-quenched condition up to 500 °C, causing a decrease in hardness. In addition, the most significant hardness drop up to 500 °C was observed between 330 and 500 °C where stage III takes place and the majority of the available carbon was found in cementite, as shown in Fig. 7(b).

For the high temperature tempering range, the onset of cementite dissolution and alloy carbide precipitation was determined as 500 °C, thus, the peak hardening was expected at about 575 °C based on the increase in the derivative signal in Fig. 2(b). The peak hardening temperature was confirmed with hardness measurements and is shown in Fig. 4 as a stabilization of the hardness levels between 500 and 575 °C. Mössbauer spectroscopy confirms cementite dissolution between 500 and 650 °C based on the decreased carbon amount in cementite within this temperature range as shown in (Fig. 7(b)). However, no confirmation of carbon return to solution is presented since lattice parameter analysis using XRD did not provide significant differences in the same temperature range. The metastable behavior of cementite with respect to alloy carbides is the driving force for cementite dissolution. Thus, the extra carbon provided by cementite may be utilized for alloy carbide precipitation, not necessarily returning to solution during this process. However, Ustinovshchikov’s^23^ results showed a momentary increase in ferritic lattice parameter associated with cementite dissolution during the secondary hardening process, which would result in an expansion as observed in the dilatometric measurements in this study. TEM CDF micrographs confirmed the low precipitation of alloy carbides at 500 °C and significantly greater amounts at 575 °C. Considerable recovery also takes place at these temperatures as demonstrated by the dislocation density analysis through XRD (Fig. 8(b)), which could be responsible for the release of carbon trapped at dislocations, another source of carbon for alloy carbide precipitation. Overall, the increase in the derivative signal between 500 and 575 °C was attributed to alloy carbide precipitation.

An increase in temperature from 575 to 650 °C caused a decrease in the derivative signal. Mössbauer spectroscopy showed that enrichment of Mn in cementite occurs, reducing the ferrite and cementite unit cell volumes^29^. Due to the increased precipitation at about 575 °C, recovery became slow, likely related to carbides pinning the dislocations and thus the drop in electrical resistivity observed in the same temperature range was associated with carbon and alloying elements leaving solution, *i*.*e*. Mn segregation to cementite and perhaps some additional precipitation of alloy carbides.

Finally, the reason behind the increase in the dilatometer derivative signal observed from 650 to 700 °C remains unclear since electrical resistivity associated with dislocation density analysis suggested the return of alloying elements to solution whereas a lack of change in B_hf_(α), α(1), and α(2 + C) in the same temperature range indicated that no alloying elements or carbon partitioning between the ferritic matrix and the carbides takes place. However, it is hypothesized that the increase could also be due to the reduction in the rate of Mn segregation to cementite, which is initiated at 575 °C and reaches a maximum at 650 °C. It is possible that two factors may control the Mn segregation to cementite, namely diffusivity and solubility. In the early stages of Mn segregation, diffusivity is likely responsible for the rate of segregation since only a para-equilibrium amount of Mn is expected in cementite. With increased temperatures, Mn diffusivity increases and a maximum rate of segregation is reached. At this stage, cementite displays elevated levels of Mn and thus solubility controls the segregation rate, since Mn atoms will have significant mobility at these temperatures. As a consequence, the segregation process slows down, causing an increase in the derivative signal.

## Conclusions

A new approach for non-isothermal tempering analysis utilizing dilatometry was proposed and carried out with a medium carbon steel. The method includes a second non-isothermal step performed with the same heating rate used for the first step in order to create a baseline for analysis. At low temperature tempering, dilatometry analysis identified carbon segregation and clustering as well as retained austenite decomposition and cementite precipitation. Secondary hardening and Mn partitioning to cementite were characterized at high temperature tempering.

Mössbauer spectroscopy confirmed the presence of tempering stages II and III, which demonstrated significant overlap, whereas most transition carbides were formed by auto-tempering and no indication of stage I was apparent. Electrical resistivity measurements associated with dislocation density analysis and TEM CDF imaging connected the expansion observed in dilatometry between 500 and 575 °C to secondary hardening or stage IV. Cementite dissolution also seems to have a contribution to the observed expansion as indicated by Mössbauer spectroscopy. Finally, Mössbauer spectra demonstrated the presence of the non-magnetic cementite phase for treatments performed up to 575 °C and higher temperatures due to the segregation of Mn from ferrite to cementite.
